# microRNAs for qPCR Normalization Under Morphofunctional Conditions in Bovine Sperm (*Bos taurus*)

**DOI:** 10.1002/mrd.70045

**Published:** 2025-08-06

**Authors:** Lucas Petitemberte de Souza, Leandro Silva Nunes, Luana Carla Salvi, Laís dos Santos Gonçalves, Luana Ferreira Viana dos Reis, Izani Bonel Acosta, Carine Dahl Corcini, Antonio Sergio Varela Junior, Fábio Gularte Barreto, Marcelo Brandi Vieira, Diego Corrêa Silveira, Jeaniffer Melgarejo Vieira, Gustavo Freitas Ilha, William Borges Domingues, Vinicius Farias Campos

**Affiliations:** ^1^ Laboratório de Genômica Estrutural, Programa de Pós‐Graduação em Biotecnologia, Centro de Desenvolvimento Tecnológico Universidade Federal de Pelotas Pelotas Brasil; ^2^ Faculdade de Veterinária Universidade Federal de Pelotas Pelotas Brasil; ^3^ Instituto de Ciências Biológicas Universidade Federal do Rio Grande Rio Grande Brasil; ^4^ PROGEN Inseminação Artificial Ltda Dom Pedrito Brasil; ^5^ Select Sires do Brasil Genética Ltda Porto Alegre Brasil

**Keywords:** *Bos* taurus, normalizing microRNAs, qPCR, semen, stability

## Abstract

Cattle represents one of the most common and widely distributed categories of large ruminants, with well‐established production practices. Fertility is a key factor that significantly influences the success of this production. Studies have shown that microRNAs (miRNAs) present in sperm cells play a crucial role as regulators of processes related to sperm functionality. miRNAs quantification by qPCR is one of the most accurate and straightforward methods, but this technique requires data normalization, and there is no universal consensus on which miRNAs should be used. The present study aimed to identify suitable miRNAs normalizers for qPCR analysis of *Bos taurus* semen. To achieve this, normalization candidates were assessed under different semen quality conditions, considering sperm morphology and motility. A small nuclear RNA (U6) and six miRNA candidates (Let‐7c‐5p, miR‐100‐5p, miR‐25‐3p, miR‐26a‐5p, miR‐204‐5p, miR‐92a‐3p) were selected. The expression stability of each candidate was analyzed using four independent methods (delta Ct, geNorm, NormFinder, and BestKeeper), under the semen quality conditions. Additionally, a comprehensive stability analysis was conducted using RefFinder, for each condition individually and for the combined conditions. The results indicated that miR‐92a‐3p was the most stable reference miRNA for motility‐related analyses, while Let‐7c‐5p emerged as the best candidate for morphology‐focused analyses. As a normalizer to analyze samples concomitantly, Let‐7c‐5p was identified as the optimal normalizer, while miR‐26a‐5p was the least stable candidate. This study provides the first identification of miRNA normalizers for qPCR analysis of *Bos taurus* semen, enabling more accurate miRNA quantification in this biological matrix and species.

## Graphical abstract

Identification of the most stable microRNAs in *Bos taurus* semen for qPCR analysis under sperm motility and morphology conditions.

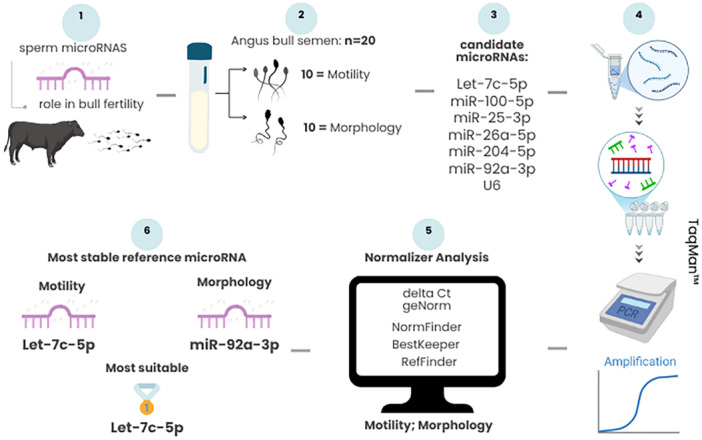

## 1 Introduction

According to the Food and Agriculture Organization of the United Nations, cattle, encompassing the species *Bos taurus*, represent the most common and widely distributed category of large ruminants, with husbandry practices established globally. There are an estimated 1.5 billion cattle worldwide, with livestock areas supporting densities of more than 250 animals per square kilometer (Gilbert et al. 2018; Food and agriculture organization of the United Nations, FAO 2018). One of the factors that most significantly impacts this production is fertility, leading to substantial economic losses for the industry. Thus, a significant percentage of reproductive failure is attributed to bull subfertility, primarily due to low or poor semen quality, which includes parameters such as sperm motility and morphology (Butler et al. 2020). Studies have shown that microRNAs (miRNAs) in sperm cells act as key regulators of various processes associated with sperm functionality. Additionally, these miRNAs hold potential as biomarkers for predicting the reproductive capacity of bulls (Bollwein and Malama 2023; Kasimanickam and Kasimanickam 2024; Santiago et al. 2022).

MiRNAs are small noncoding RNAs approximately 22 nucleotides long, characterized by multistep biogenesis and a complex posttranscriptional regulatory mechanism. Their gene expression modulation occurs, between miRNAs and target mRNAs, through interactions associated with molecular mechanisms that include translation inhibition or the promotion of mRNA degradation (Nalbant and Akkaya‐Ulum 2024; Shang et al. 2023; Suzuki 2023).

Sperm miRNAs have been highlighted for over a decade as potential biomarkers for bull fertility (de Souza et al. 2024; Govindaraju et al. 2012; Salas‐Huetos et al. 2023; Ugur et al. 2022) According to Faraldi et al. (2018), miRNAs display all the essential characteristics to be an accurate biomarker, such as sensitivity, specificity and non‐invasiveness. Furthermore, the chosen technique for quantifying different expression profiles must demonstrate high sensitivity, specificity, and reproducibility, such as quantitative PCR (qPCR) assay (Faraldi et al. 2018). Thus, bull sperm miRNAs quantification can be easily achieved by using the qPCR technique (Alves et al. 2019; Alves et al. 2021; dos Santos da Silva et al. 2021; Fagerlind et al. 2015; Kasimanickam et al. 2022; Menezes et al. 2020).

The qPCR technique requires data normalization, which involves comparing the expression level of a target miRNA to that of a reference miRNA with stable expression unaffected by varying experimental conditions (Blödorn et al. 2023). However, data normalization is a controversial issue, as there are no universally accepted normalizing miRNAs (Faraldi et al. 2018). Furthermore, Mohammadian et al. (2013) elucidate that the choice of different normalization strategies can affect the outcomes of miRNA expression analysis. Therefore, it becomes important to select the most appropriate normalizing miRNAs for each condition. Previous studies have identified reference miRNAs for analyzing circulating miRNA expression in bovine serum (Bae et al. 2015). In the same sense, Mahdipour et al. (2015) identified and validated miRNAs as normalizers for qPCR data for bovine embryos and oocytes. Currently, there are no miRNAs validated to normalize qPCR data to identify differential expression of bovine sperm miRNAs. Some studies normalize these qPCR data using global mean expression (Fagerlind et al. 2015), with genes such as PPIA (Singh et al. 2023), as well as other small noncoding RNA molecules, such as the small nuclear RNA U6 (Menezes et al. 2020) or miRNAs, as miR‐99b and miR‐425‐5p, used as normalizers for other tissues and conditions (Alves et al. 2019, 2021). However, as previously demonstrated by our research group, the selection of an inappropriate reference miRNA profoundly impacts the results, leading to erroneous interpretations of biological data (Blödorn et al. 2023; Martins et al. 2024; Pagano et al. 2024).

To utilize sperm miRNAs as fertility biomarkers in bulls, it is essential to prospect a universally accepted methodology that enables the normalization of qPCR data in bovine sperm miRNA. Therefore, the aim of the present study is to identify and validate the best reference miRNAs for bovine sperm related to different profiles of sperm motility and morphology in *Bos taurus*.

## 2 Methods

### 2.1 Ethics and Animal Welfare

Fresh semen samples were obtained from Angus bulls, provided by Brazil's Select Sires LTDA through a partnership agreement with the Federal University of Pelotas. The experimental protocols were conducted in accordance with ethical research guidelines and animal care standards, receiving approval from the Animal Ethics Committee of UFPel under process number 23110.037079/2024‐68. Additionally, the principles established by the Brazilian College of Animal Reproduction and the International Guiding Principles for Biomedical Research Involving Animals (Society for the Study of Reproduction) were adhered to.

### 2.2 Semen Samples

The semen samples were collected using the artificial vagina method. Following collection, each semen sample was diluted at a 1:1 (v/v) ratio with the commercial diluent BoviFree (Minitube, Germany). Subsequently, the samples were transported to the laboratory under controlled temperatures (4°C–10°C) for motility assessment, sperm functional morphology analysis, and microRNA isolation.

### 2.3 Experimental Design

Twenty bulls (*n* = 20) were selected for this study based on sperm motility and morphology parameters. Further analysis was conducted to identify the most suitable reference sperm miRNAs for normalization. Ten bulls were classified as having the highest sperm motility and morphology values, while the remaining 10 were categorized as having the lowest values.

#### 2.3.1 Sperm Morphology

The standard fixed unstained wet mount method was used for sperm morphology assessment, according to (Freneau et al. 2010). Sperm morphology was evaluated by analyzing a minimum of 100 spermatozoa per sample using differential interference contrast microscopy on a thin cover‐slip preparation of fresh semen.

For group definition, the following morphological parameters were analyzed immediately after collection: acrosomal abnormalities, head defects, presence of vacuoles, proximal cytoplasmic droplet (PCD), damage to the midpiece (major defects), detached head, and bent tail (minor defects). The criteria for rejection included major defects exceeding 20% or total abnormalities (major and minor defects combined) exceeding 30%. At the end of the analyses, five samples were obtained for each experimental group (approved or nonapproved).

#### 2.3.2 Sperm Motility and Kinetics

For the analysis of sperm motility and kinetics parameters, the Computer Assisted Sperm Analysis (CASA) system (AndroVision Minitube, Germany) was used, according to (O'Meara et al. 2022). Approximately 2.000 spermatozoa were analyzed across up to 10 fields per sample. The analyzed parameters included: total motility (%), progressive motility (%), mean path distance (DAP, μm), curvilinear distance (DCL, μm), straight‐line distance (DSL, μm), average path velocity (VAP, μm/s), curvilinear velocity (VCL, μm/s), straight‐line progressive velocity (VSL, μm/s), straightness (STR, % = VSL/VAP × 100), linearity (LIN, % = VSL/VCL × 100), wobble coefficient (WOB, % = VAP/VCL × 100), lateral head displacement amplitude (ALH, μm), and beat‐cross frequency (BCF, Hz). The motility analyses were used to define five samples for each experimental group (low/moderate or High).

#### 2.3.3 Candidate miRNAs for Data Normalization

This study included six miRNAs and U6 as potential normalizers for qPCR assays using bovine sperm. The selection of these targets was based on previous research that identified normalizers for semen in other species. Specifically, miRNAs −92a‐3p and −100‐5p were chosen as they have been identified as normalizers for sperm miRNA in *Homo sapiens* (Corral‐Vazquez et al. 2017). The let‐7c was also considered a candidate (Zhang et al. 2015), and miR‐25 (Pu et al. 2022) has been used as a normalizer for swine (*Sus scrofa*). Additionally, miR‐204‐5p was selected based on the “*A technical guide to identifying miRNA normalizers using TaqMan Advanced miRNA Assays,*” recommended for testis samples. The probes used in this study were TaqMan Advanced miRNA and, although they are designed for human species, the mature miRNA sequence exhibits 100% homology with *Bos taurus* (Table 1).

**Table 1:** List of Taqman advanced miRNA probes for specific cDNA synthesis and qPCR.

Assay name	Assay ID	Mature miRNA sequence
hsa‐let‐7c‐5p	478577_mir	UGAGGUAGUAGGUUGUAUGGUU
hsa‐miR‐100‐5p	478224_mir	AACCCGUAGAUCCGAACUUGUG
hsa‐miR‐204‐5p	478491_mir	UUCCCUUUGUCAUCCUAUGCCU
hsa‐miR‐25‐3p	477994_mir	CAUUGCACUUGUCUCGGUCUGA
hsa‐miR‐26a‐5p	477995_mir	UUCAAGUAAUCCAGGAUAGGCU
hsa‐miR‐92a‐3p	477827_mir	UAUUGCACUUGUCCCGGCCUGU
U6 snRNA	001973	GTGCTCGCTTCGGCAGCACATATACTAAAATTGGAACGATACAGAGAAGATTAGCATGGCCCCTGCGCAAGGATGACACGCAAATTCGTGAAGCGTTCCATATTTT (Control sequence)

### 2.4 RNA Isolation

Total RNA extraction from spermatozoa was performed by combining the use of TRIzol reagent (Invitrogen, USA) and the PureLink RNA kit (Invitrogen, USA), with minor adaptations made by our research group (Domingues et al. 2020). The total concentration of sperm cells used was 10 × 10^6^ measured in a Neubauer chamber. In brief, Lysis Buffer and TRIzol reagent were added to the semen samples, followed by vortexing. Mechanical lysis was performed using a 26‐gauge needle. Subsequently, 200 µL of chloroform was added to the samples. After vigorous shaking and incubation for 5 min at room temperature, the mixture was centrifuged at 12,000 × g at 4°C for 20 min. After centrifugation, the upper aqueous phase was transferred to a new microcentrifuge tube, and ethanol was added. The samples were transferred to a column from the PureLink RNA kit. The RNA was bound to the membrane of the spin column and subsequently washed using specific buffers by centrifugation. Thereafter, the RNA was eluted in pre‐heated 30 µL RNase‐free water. The concentration and purity of the RNA was determined using a UV spectrophotometer. All the RNA samples were stored at 80°C until further use.

### 2.5 cDNA Synthesis and TaqMan qPCR

The cDNA synthesis of the target miRNAs was performed using the TaqMan Advanced miRNA cDNA Synthesis Kit (Applied Biosystems, Waltham, MA, USA), following the manufacturer's instructions. All cycling steps were carried out on the SimpliAmp Thermal Cycler (Applied Biosystems, Waltham, MA, USA), and the samples were stored at −20°C for subsequent qPCR analysis.

For qPCR assay, the TaqMan Fast Advanced Master Mix (Applied Biosystems, Waltham, MA, USA) was used, following the manufacturer's protocol. The thermal cycling conditions for RT‐qPCR were as follows: enzyme activation at 95°C for 20 s (1 cycle), followed by denaturation at 95°C for 1 s and annealing/extension at 60°C for 20 s, both repeated for 40 cycles. Samples were assayed in triplicates using the QuantStudio 3 qPCR machine (Applied Biosystems, USA).

### 2.6 Normalizer Analysis

Four algorithms were used to analyze the stability of each candidate. The comparative delta‐Ct method compares the mean standard deviation of the Ct values for each candidate across all combinations (Silver et al. 2006). BestKeeper, based on the individual stability of the mean absolute deviation of raw Cp values, was also employed (Pfaffl et al. 2004). NormFinder calculates intra‐ and intergroup variations (Andersen et al. 2004). In contrast, geNorm uses the mean standard deviation in pairwise comparisons with all other candidates as a measure of stability (Vandesompele et al. 2002).

To combine the rankings from these four algorithms, RefFinder provides a geometric meaning of these rankings (Xie et al. 2012), and the RefSeeker R package was used as a tool to construct a comprehensive consensus ranking of stability across all methods, as described by (Petersen et al. 2023).

### 2.7 Statistical Analysis

An unpaired *t*‐test was performed for each sperm quality condition (motility and morphology), and the results were expressed as mean ± standard error of the mean (SEM). The motilities were allocated into quartiles, with the lowest 25% defined as bulls with low or moderate motility, and those above the 75% considered as bulls with high motility. For the motility and sperm kinematics parameters, a statistical significance value was defined when *p* < 0.05. The statistical significance of the difference between the groups, as well as the group definition graphs and normalizer analyses, were performed using GraphPad Prism version 8.0.1 (GraphPad Software, USA).

## 3 Results

### 3.1 Morphological Parameters

Among the seven sperm morphological parameters evaluated, defects in the intermediary piece were the only abnormalities observed to be more prevalent in the Approved group. However, when considering the major defect rate, the Non‐approved group exhibited a rate of 24%, while the Approved group had a rate of 12.8%. Regarding total pathologies, the Approved group exhibited a rate of 19%, whereas the unapproved group had an average of 50.20%. As for specific defects in the sperm head, neither group showed a defect rate above 10% (Table 2).

**Table 2:** Evaluation of major defects and total pathologies in morphological quality parameters in *Bos taurus* semen samples.

Groups	Across. (%)	Head abnor. (%)	Vacuoles (%)	PCD (%)	Intermed. piece (%)	Major defects (%)	Isolated head (%)	Folded tail (%)	Minor defects (%)	Total pathol. (%)
Approved	3.20 ± 1.02	3.20 ± 0.73	2.80 ± 0.80	1.80 ± 0.80	1.20 ± 0.58	**12.20** ± **2.28**	2.40 ± 0.81	8.00 ± 2.04	10.40 ± 2.24	**22.60** ± **2.20**
Nonapproved	3.40 ± 0.67	6.00 ± 1.82	5.80 ± 1.15	7.60 ± 3.85	0.40 ± 0.24	**24.00** ± **4.88**	2.40 ± 0.40	23.80 ± 6.59	26.20 ± 6.59	**50.20** ± **2.08**

*Note:* Data are expressed as means ± standard error of the mean (SEM). To be considered failed, the samples must have major defects exceeding 20% or total abnormalities (major and minor defects combined) exceeding 30%. Across = acrosomal abnormalities; Head abnor. = head defects; Vacuoles = presence of vacuoles; PCD = proximal cytoplasmic droplet; Intermed. piece = damage to the midpiece; Isolated head = detached head; and Folded tail = bent tail.

### 3.2 Sperm Motility and Kinetics

**Figure 1:**
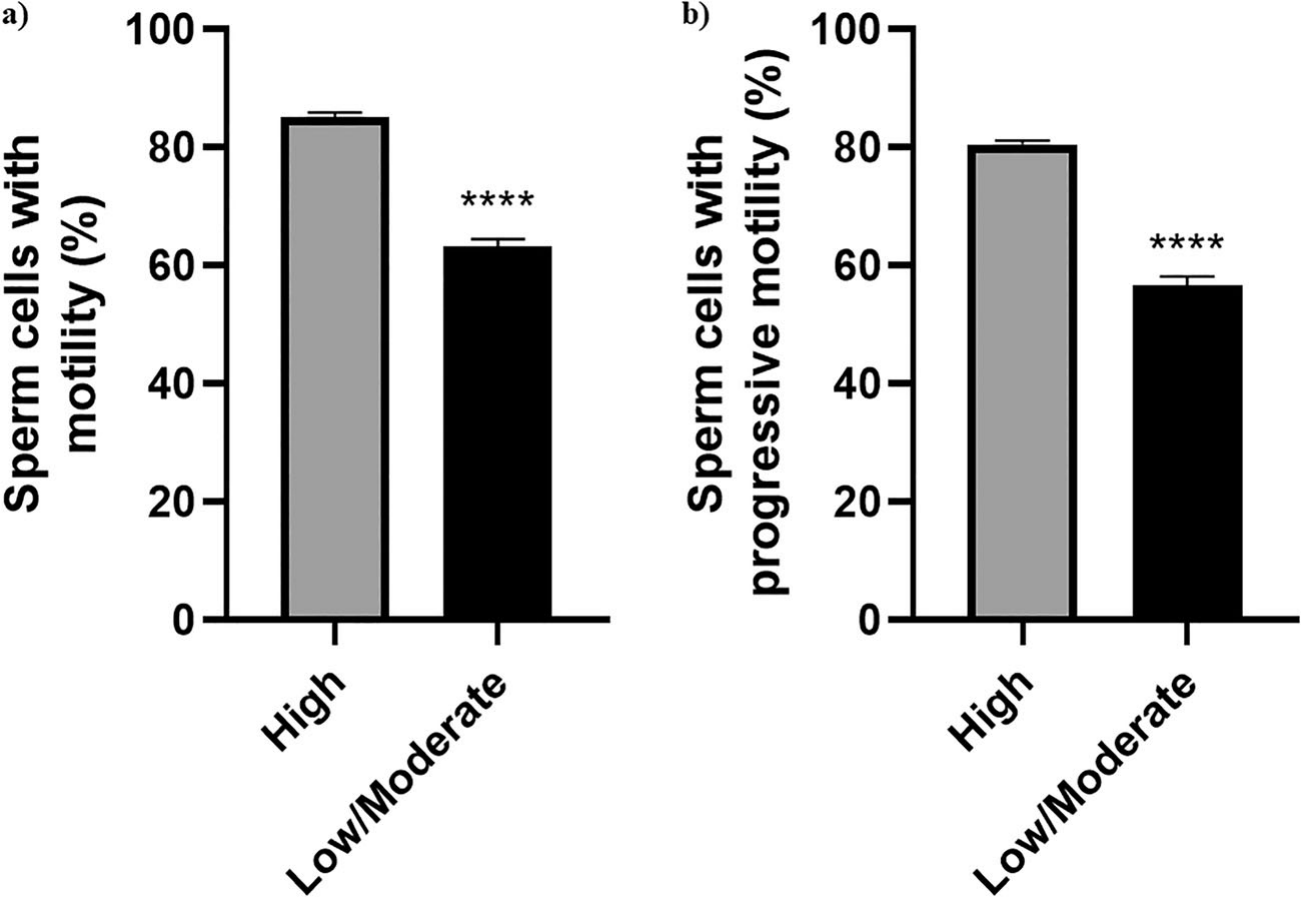
Total and progressive motility in *Bos taurus* sperm cells. Results are expressed as the mean and standard error of the mean (SEM) for each group (unpaired *t*‐test). **** = *p* < 0.0001. (a) Total motility = 85.15% (Hight) and 63.19% (Low/Moderate). (b) 80.47% (High) and 56.54% (Low/Moderate).

According to the CASA system for sperm evaluation, a significant difference was observed in sperm motilities between the High and Low/Moderate groups (Figure 1). Total motility in the High group was 85.15% while that in the Low/Moderate group was 63.19% (Figure 1A). Similarly, progressive motility was 80.47% in the High group and 56.54% in the Low/Moderate group (Figure 1B). Furthermore, among the kinetic parameters analyzed, except for DSL, WOB and BCF, there were no significant differences between the High and Low/Moderate motility groups (Table S1).

### 3.3 Normalizer: Morphology and Motility

**Figure 2:**
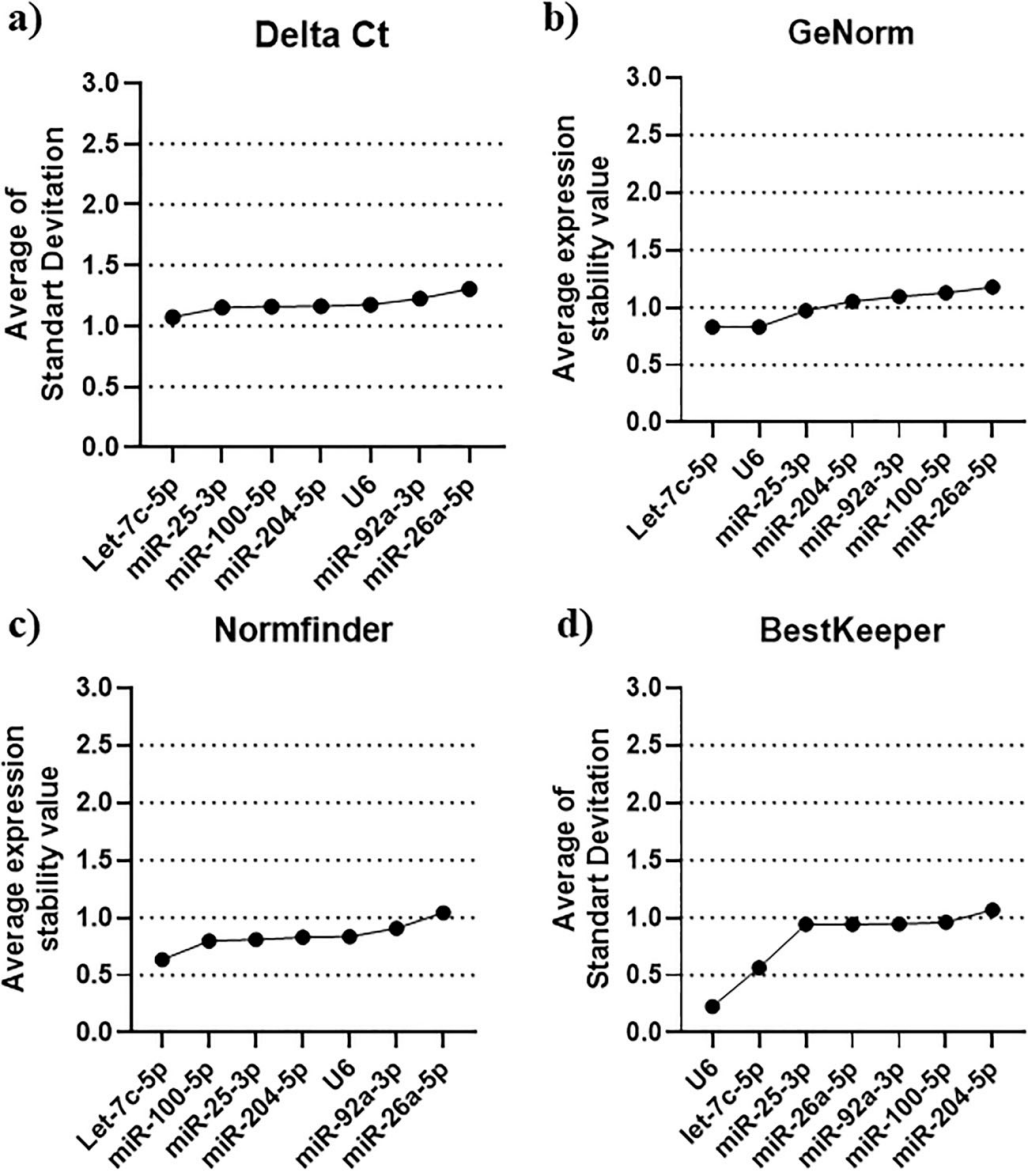
Overall stability scores of the seven data‐normalizing microRNA candidates for *Bos taur*us semen with different sperm motility profiles. Scores are provided by the delta Ct (a), geNorm (b), NormFinder (c), and BestKeeper (d) algorithms.

**Figure 3:**
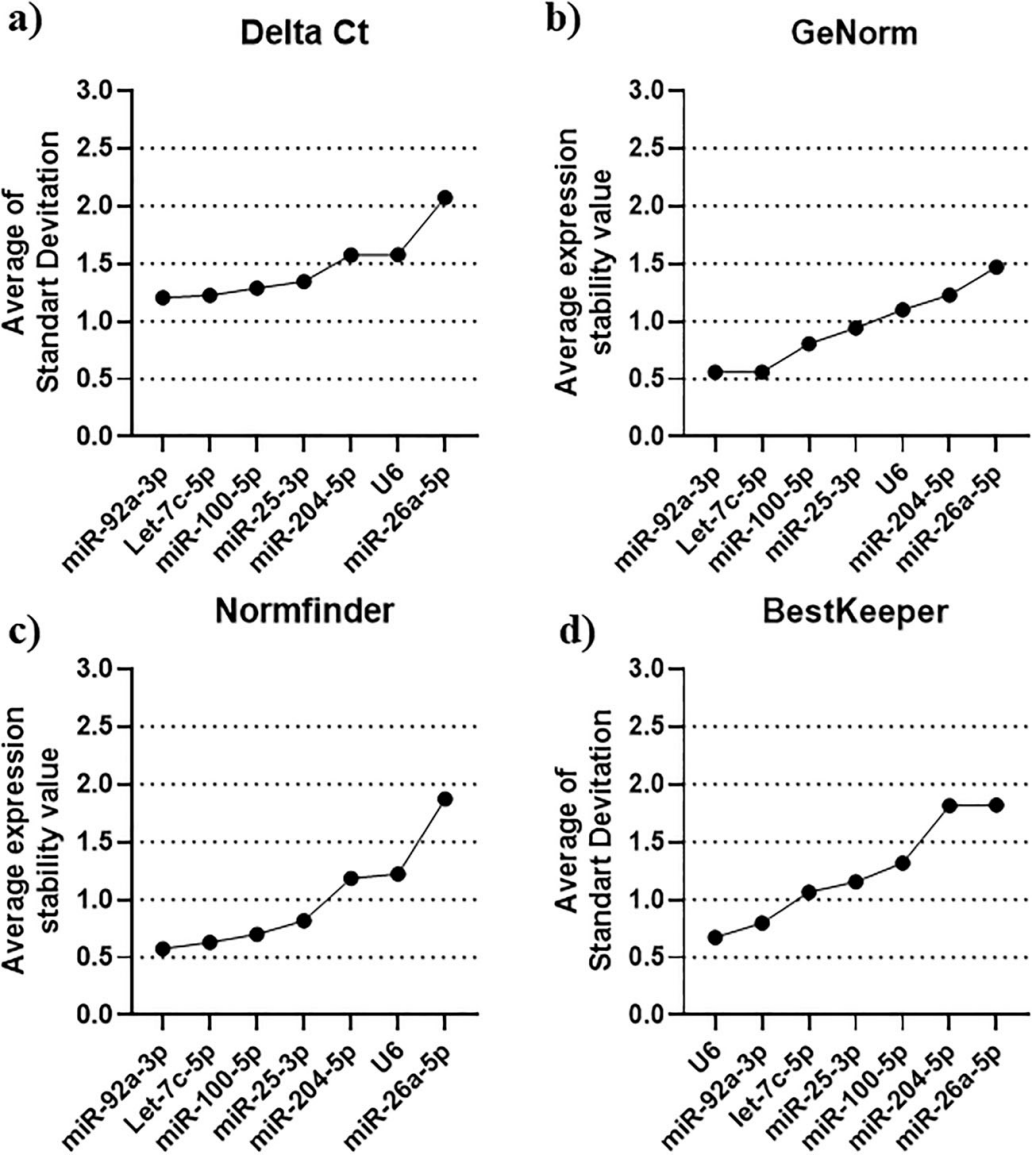
Overall stability scores of the seven data‐normalizing microRNA candidates for *Bos taurus* semen with different sperm morphology profiles. Scores are provided by the delta Ct (a), geNorm (b), NormFinder (c), and BestKeeper (d) algorithms.

Stability analyses were conducted for the candidate miRNAs and U6 across four algorithms (Delta‐Ct comparative, BestKeeper, NormFinder, and geNorm) according to different bull sperm quality profiles (morphology or motility). It was observed that each algorithm yielded a distinct ranking of stability among the targets analyzed for motility profiles (Figure 2) and morphology profiles (Figure 3). Complementarily, the means and standard deviation of the Cycle Threshold (Ct) of RT‐qPCR of the candidate reference miRNAs tested under motility (Table S2) and sperm morphology (Table S3) conditions were calculated. None of the candidate reference miRNAs showed statistical difference in the comparison between the groups.

#### 3.3.1 Comparative Delta‐Ct

The stability of the reference miRNA expression, assessed using the comparative delta‐Ct method, exhibited the following classification when analyzing the motility profiles of bull spermatozoa: Let‐7c‐5p (1.07) > miR‐25‐3p (1.15) > miR‐100‐5p (1.16) > miR‐204‐5p (1.16) > U6 (1.17) > miR‐92a‐3p (1.27) > miR‐26a‐5p (1.30) (Figure 2A). In contrast, when evaluating the sperm morphology profiles using the same analytical method, the stability classification was as follows: miR‐92a‐3p (1.20) > Let‐7c‐5p (1.22) > miR‐100‐5p (1.28) > miR‐25‐3p (1.34) > miR‐204‐5p (1.57) > U6 (1.57) > miR‐26a‐5p (2.07) (Figure 3A).

#### 3.3.2 geNorm

The geNorm analysis generates pairwise comparison values for all candidates as a measure of stability. As with the other algorithms, lower values indicate more stable reference miRNAs. For sperm motility analysis, the stability ranking was as follows: Let‐7c‐5p (0.83), U6 (0.83) > miR‐25‐3p (0.97) miR‐204‐5p (1.05) > miR‐92a‐3p (1.09) > miR‐100‐5p (1.13) > miR‐26a‐5p (1.18) (Figure 2B). In the geNorm analysis of sperm morphology differences, the stability ranking was: miR‐92a‐3p (0.52), Let‐7c‐5p (0.52) > miR‐100‐5p (0.80) > miR‐25‐3p (0.94) > U6 (1.10) > miR‐204‐5p (1.12) > miR‐26a‐5p (1.47) (Figure 3B).

#### 3.3.3 NormFinder

The NormFinder algorithm assessed the most stable reference miRNAs by expressing the average stability values of their expression. When analyzing the data from the motility groups, the following ranking was observed: Let‐7c‐5p (0.63) > miR‐100‐5p (0.79) > miR‐25‐3p (0.81) > miR‐204‐5p (0.83) > U6 (0.83) > miR‐92a‐3p (0.90) > miR‐26a‐5p (1.20) (Figure 2C). The same analysis, applied to samples with morphological differences, ranked the candidate miRNAs as follows: miR‐92a‐3p (0.57) > Let‐7c‐5p (0.63) > miR‐100‐5p (0.70) > miR‐25‐3p (0.81) > miR‐204‐5p (1.18) > U6 (1.24) > miR‐26a‐5p (1.87) (Figure 3C).

#### 3.3.4 BestKeeper

The stability analyses of the targets performed by the BestKeeper algorithm evaluated different result formats, including correlation coefficients, covariance percentages, and the mean of the standard deviations. Therefore, considering that the final consensus ranking is constructed using RefFinder, the results from BestKeeper were classified based on this value. Regarding the analysis of normalizers under the sperm motility condition, the following stability ranking was observed: U6 (0.22) > Let‐7c‐5p (0.56) > miR‐25‐3p (0.94), miR‐26a‐5p (0.94) > miR‐92a‐3p (0.94), miR‐100‐5p (0.96) > miR‐204‐5p (1.06) (Figure 2D). When analyzing the candidates with BestKeeper under different sperm morphology profiles, the following stability ranking was found: U6 (0.67) > miR‐92a3p (0.79) > Let‐7c‐5p (1.06) miR‐25‐3p (1.15) > miR‐100‐5p (1.38) > miR‐204‐5p (1.81) > miR‐26a‐5p (1.82) (Figure 3D).

#### 3.3.5 RefFinder

**Figure 4:**
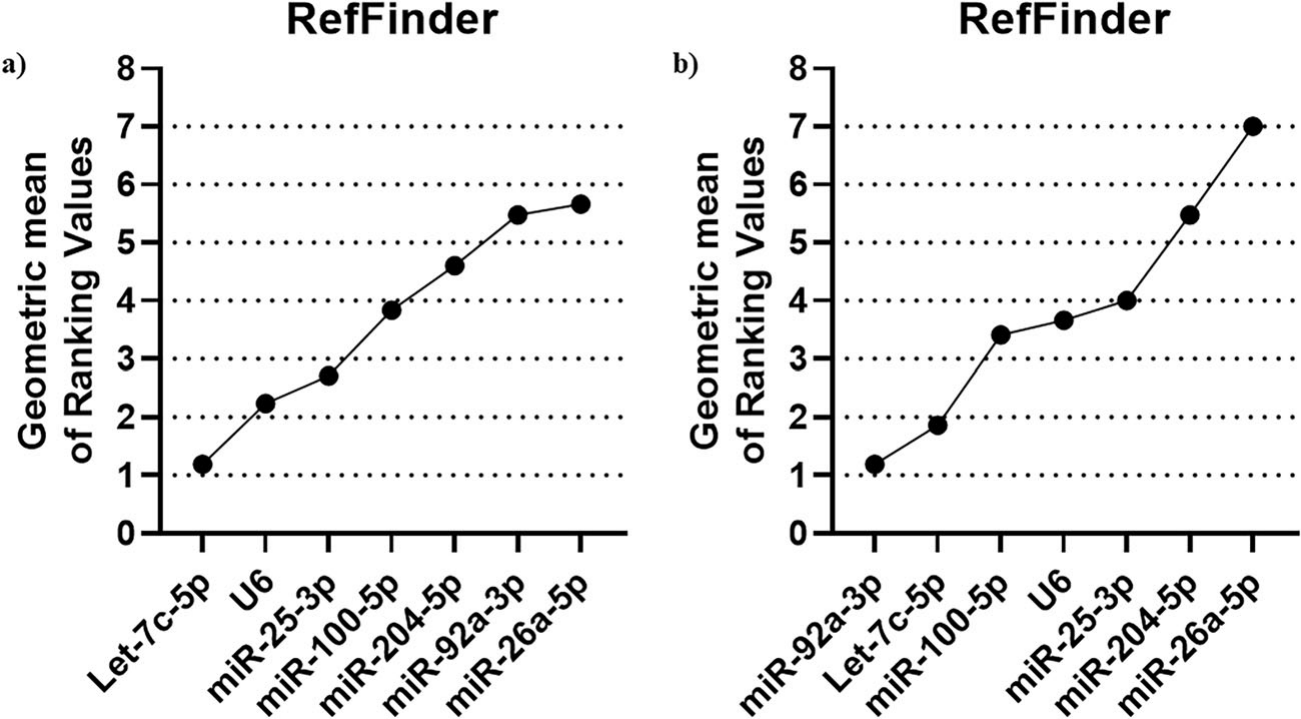
Comprehensive consensus stability score of the seven data normalization microRNA candidates for *Bos taurus* semen. The comprehensive score was performed by RefFinder based on previous scores provided by the delta Ct method, geNorm, NormFinder, and BestKeeper. (a) RefFinder stability score in samples from the Motility groups. (b) RefFinder stability score in samples from the Morphology groups.

The RefSeeker R package, through its RefFinder tool, performs a ranking analysis to determine the comprehensive consensus on the stability of the analyzed candidates across all four algorithms. Thus, RefFinder assigns individual weights to each normalization candidate based on the rankings of the methods analyzed above. Through the calculation of the geometric mean, the candidates that had the lowest means, and consequently the most stable, were: Let‐7c‐5p (1.18) > U6 (2.23) > miR‐25‐3p (2.71) > miR‐100‐5p (3.83) > miR‐204‐5p (4.60) > miR‐92a‐3p (5.47) > miR‐26a‐5p (5.66) for motility (Figure 4A). The same calculation was performed to assign the best candidates based on the set of previous stability classifications for sperm morphology analyses, with the following results: miR‐92a‐3p (1.18) > Let‐7c‐5p (1.86) > miR‐100‐5p (3.40) > U6 (3.63) > miR‐25‐3p (4) > miR‐204‐5p (5.47) > mir‐26a‐5p (7) (Figure 4B).

#### 3.3.6 Reference miRNAs for Conditions Analyzed Conjointly

**Figure 5:**
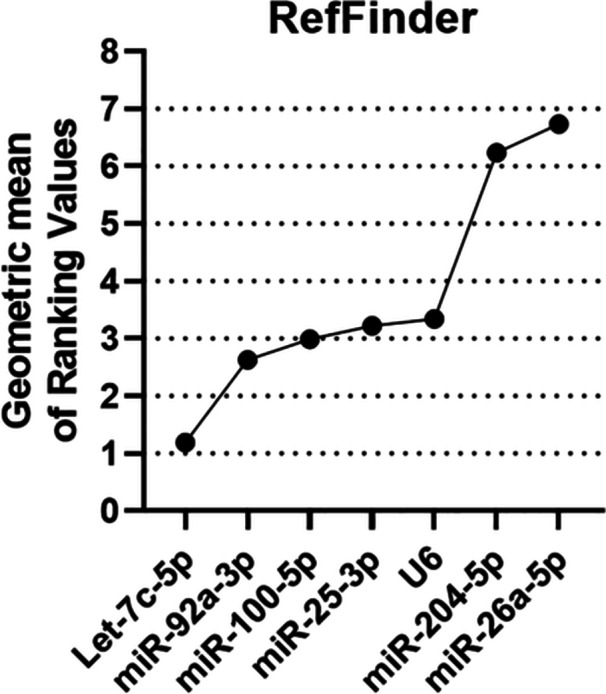
Comprehensive consensus stability classification of the seven microRNA candidates for data normalization to *Bos taurus* semen to jointly analyze analyzed sperm quality variables (motility and morphology). Comprehensive ranking was performed by RefFinder based on previous rankings provided by delta Ct method, geNorm, NormFinder and BestKeeper.

When grouping all the samples with different seminal qualities, both by motility and morphology profiles, the RefFinder ranked the candidate reference genes based on their stabilities. The most stable candidates for use in data normalization concerning the seminal quality of bulls were: Let‐7c‐5p (1.18) > U6 (2.63) > miR‐100‐5p (2.99) > miR‐25‐3p (3.22) > miR‐204‐5p (3.34) > miR‐92a‐3p (6.23) > miR‐26a‐5p (6.73) (Figure 5).

## 4 Discussion

As far as we know, there are no studies that have identified the stability of reference miRNAs for data normalization by qPCR for miRNA expression analysis in bull semen. Therefore, this study explored the stability of six candidate reference miRNAs and U6 in bovine sperm under different quality conditions. Indeed, qPCR has been used to investigate expression profiles of hundreds of bovine sperm miRNAs to assess potential fertility markers in these animals (de Souza et al. 2024; Salas‐Huetos et al. 2023). Furthermore, according to the guidelines of the Minimum Information for Publication of Quantitative Real‐Time PCR Experiments (MIQE), stable endogenous reference genes should be used for the normalization of target RNAs, including miRNAs. Moreover, the MIQE guidelines suggest that such references should preferably be of the same RNA type as the targets (Bustin et al. 2009).

The use of miRNAs as species‐, tissue‐, and condition‐specific normalizers is frequently highlighted in the literature. For example, this approach was demonstrated in a study by Blödorn et al. (2023) which listed reference miRNAs for *Oreochromis niloticus* under acute cold stress effects in tissues such as the brain, liver, and gills. The authors observed that miR‐103 was considered the most stable, while miR‐455 was the least stable for normalization (Blödorn et al. 2023). In contrast, the selection of reference miRNAs for *O. niloticus*, analyzing the same tissues and candidate miRNAs under osmotic stress, indicated that the combination of miR‐455 and miR‐23 is a robust reference for normalizing miRNA expression under this condition (Martins et al. 2024). Interestingly, the least stable candidate (miR‐455) for data normalization in acute cold stress analysis is the most stable for expression analysis under salinity conditions in these animals. These findings suggest that it is necessary to select different miRNAs for data normalization by qPCR for their respective analyses.

In our analysis, the most stable miRNAs for data normalization to assess sperm miRNA expression in *Bos taurus* were miR‐92a‐3p and Let‐7c‐5p for morphological and motility sperm quality conditions, respectively. Previous studies determined some reference miRNAs for sperm and semen for other species, such as *Homo sapiens* and *Sus scrofa*. For instance, miR‐100‐5p and miR‐30a‐5p are suggested for the normalization of sperm miRNA quantification in *Homo sapiens* (Corral‐Vazquez et al. 2017). In contrast, miR‐25 is the best normalizer for *Sus scrofa* semen (Pu et al. 2022) and miR‐186, miR‐23a, and miR‐27 are the most suitable references for data normalization in spermatozoa cryopreservation studies in *Sus scrofa* (Zhang et al. 2015). However, just as tissues and conditions preferably require specific reference miRNAs, species also tend to follow the same specificity regarding normalizers.

Our findings reinforce the two points outlined above: that specific normalizers are needed for different conditions within the same biological matrix, and that there are species‐specific normalizers for the same matrix. Thus, the selection of targets in this study for sperm normalization candidates in cattle was based on other candidates and sperm reference genes previously identified in other species (Corral‐Vazquez et al. 2017; Pu et al. 2022; Zhang et al. 2015). Such a choice, supported by literature, directed our study towards candidates considered to be the most stable molecules across different profiles and seminal quality conditions. Another priority was the homology of these candidate miRNAs with *Bos taurus*, the species targeted in this study (Table 1).

The small nuclear RNA U6 was the only candidate among those selected that is not a miRNA. However, its selection was based on the frequent use of this molecule to normalize data in qPCR analyses in bovines. An example is that U6 exhibited highly consistent expression across eight different tissues, being proposed as an endogenous control for heart, lung, kidney, muscle, fat, uterus, spleen, and small intestine, as reported in the findings of Li et al. (2014). However, the widespread use of U6 as a potential normalizer across different tissues cannot be considered when selecting the best data normalizer. This is because, as previously mentioned, no universal miRNA currently exist for normalizing qPCR data across different variables and expression patterns (Faraldi et al. 2018). Furthermore, our findings support the absence of a universal miRNA normalizer, as our analyses showed that U6 was not the best candidate for morphology or sperm motility conditions. It is a fact that U6 has been used to normalize semen data in bulls (Menezes et al. 2020). However, the lack of previous validations of this molecule acting as a normalizer for differentially expressed miRNAs in bull semen led us to perform confirmatory analyses of its stability.

Regarding the candidates with the best stability, our study indicated that miR‐92a‐3p outperformed the other candidates in the analyses performed on the Approved and Non‐Approved sperm morphology samples. The selection of this miRNA followed the criteria outlined above, such as homology with the bovine species and its potential for stability, based on the literature. The hsa‐miR‐92a‐3p was shown to be the most uniformly expressed molecule in human sperm (Corral‐Vazquez et al. 2017). However, the same authors found that hsa‐miR‐92a‐3p exhibited low similarity with the Mean‐Centering Restricted (MCR) method, with results not better than those obtained by U6. An interesting finding raised by Salilew‐Wondim et al. (2014) is that miR‐92a is among the 10 most predominantly abundant miRNAs in granulosa cells of subordinate and dominant follicles in *Bos taurus*. Furthermore, bta‐miR‐92a is one of the 20 most highly expressed miRNAs in extracellular vesicles (EVs) from bovine serum, and it is one of the 13 miRNAs associated with the immune system (Quan et al. 2019, 2020). The findings mentioned above regarding the uniformity and high predominance of this molecule in different biological matrices reinforce its potential role in expression normalization for possible conditions, such as sperm quality. In our study, except for BestKeeper, all other algorithms identified miR‐92a‐3p as the miRNA with the best stability for analyzing the samples regarding their sperm morphology profile.

When performing the analyses for data normalization in bull semen samples with different sperm motility profiles, let‐7c‐5p proved to be the candidate with the best stability in 3 out of the 4 algorithms. Another similarity with miR‐92a, regarding its stability as a candidate for data normalization in motility, is that let‐7c also demonstrates high expression levels in various tissues, including the testes (Huang et al. 2011). Thus, in addition to finding abundant expression in the testicular tissue, Huang et al. (2011) also observed this high expression (above 100,000 reads) of let‐7c in the ovary. It was also observed that let‐7c is one of the top 10 most expressed miRNAs in milk exosomes when analyzing sequencing data across different species (Chen et al. 2020). These findings highlight that let‐7c is a miRNA that has consistently been studied for its expression stability across different tissues and conditions. Thus, due to its stability characteristics between groups, let‐7c can frequently be used as a data normalizer for qPCR analyses of bull semen samples with high and low motility. In addition, let‐7c‐5p also proved to be the most stable candidate for data normalization when analyses were conducted jointly with the seminal quality parameters. Thus, the selection of let‐7c‐5p as a normalization of qPCR for bovine semen is equally effective in analyses that consider sperm motility parameters, as well as in analyses of samples with different sperm morphology profiles.

In contrast to the miRNAs with the most stability across motility and morphology conditions, miR‐26a was considered in our study the least stable miRNA for both variables, in all the analyzed algorithms and in the consensus ranking provided by RefFinder. The miR‐26a was a candidate for normalizing qPCR data for analyses in oocytes and embryos in bovines and pigs, being one of the most stable for pig oocytes. (Mahdipour et al. 2015). The selection of miR‐26a was based on the study of Zhang et al. (2015) that used miR‐26a as a candidate for normalizing data in cryopreserved spermatozoa of *Sus scrofa*. However, in the same study, miR‐26a showed a significant difference in expression between fresh and cryopreserved ejaculate (Zhang et al. 2015). To support this idea, there is evidence of differential expression of miR‐26a in sperm cells of bovines related to different motility profiles, as miR‐26a showed upregulation in high‐motility bovine spermatozoa (Capra et al. 2017). Moreover, miR‐26a is a miRNA that targets mRNAs involved in the PTEN pathway, which is potentially associated with cellular processes such as apoptosis, spermatogenesis, and sperm motility (Capra et al. 2017; Xu et al. 2014). Therefore, miR‐26a does not exhibit good stability characteristics to be an effective normalizer, but it still holds potential as a biomarker for sperm motility in bulls.

It is well known that miRNAs are described as potential markers of bull fertility and that semen quality parameters are closely related to male fertility (de Souza et al. 2024). These miRNAs could serve as the basis for developing a biotechnological tool capable of detecting the reproductive potential of bulls through qPCR. To achieve this, it is necessary to identify data normalizers for qPCR. In our study, as expected, a different normalizer was suggested for each semen quality condition analyzed (morphological and kinematic parameters). Thus, miR‐92a was considered the best normalizer miRNAfor semen samples with different sperm morphology profiles. On the other hand, let‐7c emerged as the best normalizer among the candidates for analyzing semen samples from bulls with different sperm motility profiles. Still, when analyzing the entire set of samples, with both semen quality parameters, we found that let‐7c is the best normalizer for fresh semen. By selecting appropriate normalization strategies and candidate miRNAs, our study contributes to refining the analytical approaches in reproductive biology, ultimately enhancing predictive models of bull fertility. Further research is needed to validate sperm miRNAs as biomarkers across different conditions to establish their broader applicability.

## Author Contributions

**Lucas Petitemberte de Souza:** conceptualization, methodology, investigation, writing – original draft. **Leandro Silva Nunes:** investigation, methodology. **Luana Carla Salvi:** investigation, methodology. **Laís dos Santos Gonçalves:** methodology, investigation. **Luana Ferreira Viana dos Reis:** investigation, methodology. **Izani Bonel Acosta:** investigation, methodology, software. **Carine Dahl Corcini:** investigation, methodology, software, formal analysis. **Antonio Sergio Varela Junior:** investigation, methodology, software, formal analysis. **Fábio Gularte Barreto:** investigation, data curation, resources. **Marcelo Brandi Vieira:** investigation, data curation, resources. **Diego Corrêa Silveira:** investigation, data curation, resources. **Jeaniffer Melgarejo Vieira:** data curation, investigation, resources. **Gustavo Freitas Ilha:** investigation, resources, data curation. **William Borges Domingues:** investigation, conceptualization, methodology, data curation, formal analysis, supervision, writing – original draft. **Vinicius Farias Campos:** conceptualization, supervision, project administration, writing – review and editing, investigation, funding acquisition.

## Ethics Statement

The collections of biological material were carried out by qualified professionals, in accordance with ethical guidelines in research and animal care standards, receiving approval from the UFPel Animal Ethics Committee.

## Conflicts of Interest

The authors declare no conflicts of interest.

## Supporting information


**Table S1:** Evaluation of kinetic parameters in quality parameters in *Bos taurus* semen samples.

**Table S2:** Evaluation of the mean Cycle Threshold (Ct) of RT‐qPCR of candidate reference miRNAs tested under sperm motility conditions in *Bos taurus* semen.

**Table S3:** Evaluation of the mean Cycle Threshold (Ct) of RT‐qPCR of candidate reference miRNAs tested under sperm morphology conditions in *Bos taurus* semen.

## Data Availability

The data that support the findings of this study are available from the corresponding author upon reasonable request.
