# Supplementary material for: microRNAs for qPCR Normalization Under Morphofunctional Conditions in Bovine Sperm (Bos taurus)
Source: Mol Reprod Dev. 2025 Aug 6;92(8):e70045. doi: 10.1002/mrd.70045 (PMC12327186; doi:10.1002/mrd.70045)
Supplement: Supplementary file 1 — Table S1: Evaluation of kinetic parameters in quality parameters in Bos taurus semen samples. [file MRD-92-e70045-s003.docx]

**Table S1.** Evaluation of kinetic parameters in quality parameters in *Bos taurus* semen samples.

| **Groups** | **DAP**  **(μm)** | **DCL (μm)** | **DSL**  **(μm)** | **VAP**  **(μm/s)** | **VCL**  **(μm/s)** | **VSL**  **(μm/s)** | **STR**  **(%)** | **LIN**  **(%)** | **WOB**  **(%)** | **ALH**  **(μm)** | **BCF**  **(Hz)** |
| --- | --- | --- | --- | --- | --- | --- | --- | --- | --- | --- | --- |
| High | 45.26  ± 1.11 | 81.25  ± 2.64 | 33.14  ± 1.19 | 102.90  ± 2.79 | 184.70  ± 6.43 | 75.48  ± 2.87 | 0.72  ± 0.012 | 0.40  ± 0.005 | 0.559  ± 0.004 | 4.43  ± 0.14 | 31.13  ± 0.45 |
| Low/  Moderate | 36.86  ± 1.94 | 67.18  ± 3.91 | 29.59  ± 1.75 | 80.87  ± 4.28 | 147.20  ± 8.61 | 64.87  ± 3.84 | 0.78  ± 0.008 | 0.43  ± 0.003 | 0.557  ± 0.006 | 3.58  ± 0.15 | 31.17  ± 0.40 |
| P value | *** | ** |  | *** | ** | * | **** | **** |  | *** |  |

Data are expressed as means ± standard error of the mean (SEM). P value (unpaired t Test): *p < 0.05; **p < 0.005; ***p = 0.001; ***p <0.001. DAP = mean path distance; DCL = curvilinear distance; DSL = straight-line distance; VAP = average path velocity; VCL = curvilinear velocity; VSL = straight-line progressive velocity; STR = straightness; LIN = linearity; WOB = wobble coefficient; ALH = lateral head displacement amplitude; BCF = beat-cross frequency.
