# Supplementary material for: microRNAs for qPCR Normalization Under Morphofunctional Conditions in Bovine Sperm (Bos taurus)
Source: Mol Reprod Dev. 2025 Aug 6;92(8):e70045. doi: 10.1002/mrd.70045 (PMC12327186; doi:10.1002/mrd.70045)
Supplement: Supplementary file 2 — Table S2: Evaluation of the mean Cycle Threshold (Ct) of RT‐qPCR of candidate reference miRNAs tested under sperm motility conditions in Bos taurus semen. [file MRD-92-e70045-s001.docx]

**Table S2.** Evaluation of the mean Cycle Threshold (Ct) of RT-qPCR of candidate reference miRNAs tested under sperm motility conditions in *Bos taurus* semen.

| **Groups** | **let-7c-5p** | **miR-100-5p** | **miR-204-5p** | **miR-25-3p** | **miR-26a-5p** | **miR-92a-3** | **U6** |
| --- | --- | --- | --- | --- | --- | --- | --- |
| High | 32.49 ± 0.53 | 28.02 ± 0.74 | 26.24 ± 1.23 | 33.74 ± 0.98 | 29.10 ± 1.07 | 27.88 ± 1.50 | 23.73 ± 0.23 |
| Low/  Moderate | 33.20 ± 0.79 | 28.98 ± 1.51 | 27.70 ± 1.21 | 34.76 ± 0.99 | 29.58 ± 1.14 | 28.13 ± 0.71 | 23.53 ± 0.28 |

Data are expressed as means ± standard error of the mean (SEM). The results did not show a statistically significant difference (p > 0.05).
