# Supplementary material for: microRNAs for qPCR Normalization Under Morphofunctional Conditions in Bovine Sperm (Bos taurus)
Source: Mol Reprod Dev. 2025 Aug 6;92(8):e70045. doi: 10.1002/mrd.70045 (PMC12327186; doi:10.1002/mrd.70045)
Supplement: Supplementary file 3 — Table S3: Evaluation of the mean Cycle Threshold (Ct) of RT‐qPCR of candidate reference miRNAs tested under sperm morphology conditions in Bos taurus semen. [file MRD-92-e70045-s002.docx]

**Table S3.** Evaluation of the mean Cycle Threshold (Ct) of RT-qPCR of candidate reference miRNAs tested under sperm morphology conditions in *Bos taurus* semen.

| **Groups** | **let-7c-5p** | **miR-100-5p** | **miR-204-5p** | **miR-25-3p** | **miR-26a-5p** | **miR-92a-3** | **U6** |
| --- | --- | --- | --- | --- | --- | --- | --- |
| Approved Morphology | 32.95 ± 0.61 | 28.86 ± 1.68 | 27.42 ± 1.75 | 34.08 ± 1.12 | 29.29 ± 1.19 | 27.88 ± 0.52 | 23.83 ± 0.77 |
| Non-Approved Morphology | 33.46 ± 1.89 | 29.58 ± 1.53 | 29.75 ± 2.08 | 35.47 ± 1.30 | 30.58 ± 2.92 | 28.27 ± 1.53 | 24.21 ± 0.90 |

Data are expressed as means ± standard error of the mean (SEM). The results did not show a statistically significant difference (p > 0.05).
